# Staying in or Dropping Out of Elite Women's Football—Factors of Importance

**DOI:** 10.3389/fspor.2022.856538

**Published:** 2022-04-14

**Authors:** Ingrid Amalie Hoftun Bjerksæter, Pål Arild Lagestad

**Affiliations:** Department of Teacher Education and Art, Nord University, Levanger, Norway

**Keywords:** female, soccer, elite level, drop out, career

## Abstract

The average age of elite women footballers in Norway is 22.7 years, significantly lower than that of elite male footballers in Norway (26.5). This study examines the factors leading to elite female footballers ending their careers at a relatively young age, and those factors influencing other female elite players to continue. Semi-structured interviews were conducted with five female elite players who had ended their career at the age of 19–24, and four who were still active (age 26–31). Based on the age of the informants who had ended their career and the informants who were still active, this study defines “young age” as <25years of age. The female elite players experiences were analyzed and discussed in relation to earlier research. The results of the study show that the same factors affecting the early end of a career, also had an impact on continuing the career in the Norwegian top league, beyond the average age. A low level of internal and external motivation, poor financial circumstances, high stress load, long injury breaks, lack of playing time and other priorities, are all factors leading elite players to end their football careers at a young age. These factors are increasingly being addressed in relation to still active elite players, and this has an impact on the length of their playing career. Like earlier research, this study indicates that the emergence of Norwegian women's football in recent years has helped to improve conditions for being female elite players in the professional leagues in Norway.

## Introduction

In 1976, women's football was officially approved as a formal sport by the Norwegian Football Federation (NFF), and for the first time women in Norway got their own official leagues and tournaments. In the years that followed interest spread among girls and women of all ages throughout the country (Goksøyr and Olstad, 2002). By 2019 there were 113,000 active girl and women football players in Norway, and NFF was actively working to promote girls' and women's football, and it has made it a priority area also for 2020–2023 (NFF, 2021).

In 2019, the NFF expressed concern about the low average age of players in the women's Norwegian top league. According to *Tinghefte* (the report from NFF's annual assembly) in 2019, players in *Toppserien* (the Norwegian top league of women's football in Norway) had an average age of 22.7 years. This low average age was clarified further by UEFA's (Union of European Football Associations) survey, showing that Norway has a smaller number of active adult players than many other countries in UEFA (NFF, 2021). It is reasonable to argue that there could be an association between the small number of active female players, and the low average age of female elite players, because of early drop out. In comparison, a cross-sectional survey from 2019 showed that the average age of male players in *Eliteserien* (the Norwegian top league of men's football in Norway) was 26.5 years, which means that men had a significantly longer career than women. The difference at almost 4 years between men and woman dropping out of elite football, is a source of concern according to the relatively few years players participate at the top level. On the basis of these findings, the leadership of NFF is concerned that the low average age may impact Norway's competitiveness in international women's football (NFF, 2021). This concern is supported by the study of Barreira (2016): “Age of Peak Performance of Elite Women's Soccer Players”, where the result showed that national teams with higher average age on their players, performed better in the London 2012 Olympic Games, than the teams with lower average age.

The theoretical framework for this study lies within hegemonic masculinity (Connell and Messerschmidt, 2005). Kaelberer (2019) argue that football represents a social sphere for the expression of hegemonic masculinity, and suggest that football continues to be described as one of the last male preserves, in which men can act out their masculinity. In this sense, football offers a space for the performance of hegemonic masculinity. Hegemonic masculinity is a relational concept that is produced as the dominant concept that legitimates inequitable hierarchical relations between men and women (Drummond, 2003; Connell and Messerschmidt, 2005; Tucker and Govender, 2017. The universal characteristics of hegemonic masculinities are related to strength, speed, muscularity, and competitiveness as being produced as dominant—characteristics that are central in football, and giving men more acknowledgment and better conditions for staying in elite level football. Female elite players players on the other hand, get poorer conditions for doing elite sport in relation to lack of financial security—leading female to prioritize education and work outside elite football play. Studies have shown that this stress may lead to drop out among female elite players (McCormack, 2011; Gammelsæter and Solenes, 2013; Brandt-Hansen and Ottesen, 2017; Grygorowicz et al., 2019; Wang, 2019; McGreary et al., 2021). As researchers, we position ourselves within a position that promote gender equality and diversity (Drummond, 2003; Paechter, 2019), argue that there is a need to challenge the hegemonic nature of masculinized PA in every arena—also in football. According to (Paechter, 2019) understanding the gender regime of any setting gives insight into power relations about how specific groups—such as female elite football players, place and position themselves.

A few earlier studies have addressed the factors affecting the career of female elite players. Brandt-Hansen et al. (2014), in their study of Danish women footballers, found that lack of motivation, injury and shortage of time were the three principal reasons for dropping out of elite football. Likewise, lack of motivation was seen by current elite players as the most likely reason for their dropping out in the future, while the opportunity to constantly improve, the joy of performing, and finding football fun and pleasurable were given as reasons for carrying on playing. External motivation, according to Gagné and Deci (2005), refers to behavior driven by the wish to achieve rewards or avoid punishment, while internal motivation arises when behavior and participation are governed by joy, interest, or excitement relating to the activity itself. Both internal and external motivation can be presumed to play a part in the ending or continuance of playing elite football. In a survey conducted by NRK in collaboration with SVT and YLE, large financial differences between male and female elite players were revealed (Lie, 2017). The figures show that a man on the national football team earns an average of 640,000 Euro more a year than a woman on the national football team. Of 13 factors potentially playing a part in motivation to play at an elite level, “money and other material benefits” showed itself to have least significance for elite players (Brandt-Hansen et al., 2014). In addition, “making money from the sport” came out last when asked what goals and ambitions they had with football. According to Brandt-Hansen et al. (2014), this is not unexpected as the possibility of earning money and living as a full-time professional is limited in Danish women's football. Brandt-Hansen and Ottesen (2017), confirmed this in a later study, in that of the 102 elite female footballers who participated in their study, only 2% had a full professional contract. The remainder were dependent on income from outside work, study grants or help from their family. Given that only a small minority had a full-time contract, Brandt-Hansen and Ottesen (2017) suggest that women's football in Denmark is seen as an amateur elite sport, lacking opportunities to pursue it full-time. In the same study, Brandt-Hansen and Ottesen found that lack of time and inadequacy of financial resources were given as factors among women footballers ending their careers.

Both Wang (2019) and Grygorowicz et al. (2019) argue that the overall stress burden on female footballers is unsustainable, emphasizing the importance of freeing up time so that players have the opportunity to train and get sufficient rest, while being able to maintain a social life outside football. In a study by McGreary et al. (2021), elite female players found it challenging to balance education, work and football, as well as having a social life. The studies of McCormack (2011); Gammelsæter and Solenes (2013) and Brandt-Hansen and Ottesen (2017) each describe the importance of education for female football players. Although sport is an unpredictable career for all involved, elite male footballers are better able to secure themselves financially via their career, whilst female players will have little to fall back on. Brandt-Hansen and Ottesen (2017), found, in their study, that another significant factor in ending a football career was the desire to establish a family and to have children, whilst Grygorowicz et al. (2019), found that injuries were a reason for drop out among female players. Sandon et al. (2015) found that women footballers carry a greater risk of cruciate ligament injuries, and that these occur at a younger age in women than in men. The study also found that fewer women than men return to football after such injuries.

In Brandt-Hansen et al.'s (2014) study, the results showed that there was a significant difference between Danish elite players who had played for the national team, and those who had not, in the extent to which they felt trusted and supported by their coach and club. Those elite players who had experience in none of the under-16, 18, and 21 national teams nor the senior team, felt, to a greater degree, that a lack of support from coach and club was a factor stopping them for playing at elite level (Brandt-Hansen et al., 2014). Johansen (2016) also found that players who had most playing time and were regularly in the starting eleven, were happy with their coach, whilst those who often served as reserves were less so. Furthermore, women more than men thought it was important that the trainer offered them recognition, and that they felt seen.

Despite some research in this field, this earlier research suggests that the factors leading elite female football players to end or to continue their careers has been an understudied field, both in Norway and internationally. Few studies have investigated the early ending of the careers of elite female footballers.

Despite some research at this field, previous researches suggests that the factors that lead to female elite players in football quitting or continuing their careers, have not been further studied, neither in Norway or internationally. Few studies have examined the early drop out of the career of female elite football players. Based on statistics on participation in various sports federations in Norway, the Norwegian Football Federation is at the top for both men and women in terms of the number of active members. Football is the sport in Norway that most Norwegians participate regardless of gender, but at the same time a sport where you experience large and visible differences between the genders. In addition, the statistics show that the difference between the average age in elite football among men and women has a difference of 3.8 years. Women stay behind here as well, just as they stay behind in the development of elite football. Historically, women's football has not lasted as long as men's football. As mentioned earlier, it was not until 1976 that women's football was recognized as a formal sport in Norway. Men's football has a history that stretches much further back in time, and this is reflected in the dominance of men's football in Norway, as in the rest of the world. As several of the presented studies has described - the conditions for female elite players have not been good enough for woman to fully invest in a football career.

The research question of this study is: Which factors lead to elite women footballers in Norway ending their career at a young age, and which factors influence those who decide to continue playing?

## Methods

### Design

The study has a qualitative design using interviews and a phenomenological-hermeneutic approach to shed light on the research question (Tjora, 2017), by exploring elite female players' opinions, attitudes and experiences regarding the factors that made them end or continue their football career. The research project has been approved by the Norwegian Centre for Research Data (NSD), and all participants gave their signed informed consent.

### Participants

The participants were recruited via a strategic selection based on their characteristics and qualifications corresponding to the research question (Thagaard, 2013). A criterion for participation was that the female players had played at least 20 games in the Norwegian top league. The website of the Norwegian Football federation (NFF, 2021), together with that of *altomfotball* (altomfotball, 2021), were used to identify them. Information about the research project and the question of participation was sent to the manager of clubs in the Norwegian top league. They sent the information further to the individual players. In all, nine players made contact, willing to participate in the study. The selection consisted of five players who had recently ended their elite football career at an age between 19 and 24 (Mean = 21, SD = 2.3), and foue players aged 26–31 who continued to play during the 2020/2021 season (Mean = 27.8, SD = 1). There are no standards for early drop out or long participation in elite football, but the cut offs seemed reasonable according to average age among female and male elite football players. These players represented different clubs in the Norwegian top league, and had played for one or more of the following clubs: Klepp, Røa, Stabæk, Kattem, Sandviken, Arna-Bjørnar, Grand Bodø, Fart, Medkila, Amazon Grimstad, Kolbotn, Vålerenga, Fløya, LSK and Trondheims-Ørn.

### Procedures

A semi-structured interview guide with open ended questions was created (Thagaard, 2013). This included questions such as; “which factor do you feel was of importance to your ending/continuing with elite football?”. However, some questions were also created based on the results of previous research. These included questions such as; “what was the importance of education in your ending/continuing with elite football?” and “what was the importance of finances in your ending/continuing with elite football?”. To test the interview guide, a pilot interview was conducted with a still active, 25-year-old elite player, playing for a Norwegian top league club. The interview was conducted face-to-face at the home of the informant and took 120 min. The pilot interview indicated that most of the questions seemed relevant. However, because of the long interview, similar-sounding questions were deleted together with leading questions and questions that seemed unlikely to yield answers to the research question. In addition, small changes were made to the order of the questions. The first four interviews were conducted face-to-face at the football clubs of the informants. Because of the Corona situation, three interviews were conducted using the Teams app, and two interviews using the telephone. The interviews lasted between 40 and 190 min, 60 min on average.

### Data Analysis

All nine interviews were transcribed out of dialect into standard Norwegian (*bokmål*) to ensure anonymity. A phenomenological-hermeneutic approach to the data was used (Thagaard, 2013). That is, it was desirable to understand phenomena and situations from the point of view of the elite players, through them describing the world around them as they had experienced it (Thagaard, 2013). According to Thagaard (2013), hermeneutics emphasizes the interpretation of a statement, and then looks for a deeper meaning content than that immediately evident. This was done via a coding process followed by a process of categorization. In the first phase, coding of all the statements made in each interview using the analytic program NVivo 12. These codes then formed the basis for the next step, the categorization of the codes by thematic relatedness, the codes not relevant to the research question being excluded. Categorization helps to structure the meaning from long interview texts, and to make it easier to find common features (Kvale et al., 2009). On account of the two different groups of informants, the codes were divided under the main categories: “reasons for” and “reasons for continuing career past average age”. The codes represented different factors influencing elite players to: end their football career; continue their football career; or to both continue and end their career. The first process resulted in a rough categorization into 28 factors divided between the two groups. In the second analytic process, the 28 factors were used as a starting point to find common features in the data material. The 28 factors were divided into six new main categories in which factors from both groups were included, with 14 factors influencing elite players either to end or to continue their football career (Table 1).

**Table 1:** Factors influencing elite players either to end or to continue their elite football career.

**Main categories**	**Sub-categories**	**Sub-categories**
	**Factors influencing ending of elite career**	**Factors influencing continuation of elite career**
Internal and external motivation	-Lack of internal and external motivation	-High internal and external motivation
Financial circumstances and total load level	-Financial worries -Little financial support from the club - Lack of facilitation to make the elite players everyday life easier and more professional -High levels of stress according to personal finances	-Financial security from jobs outside football -Professionalism of the club -Growth of women's football - Good facilitation by the club to make the elite players everyday life easier and more professional. -Lower levels of stress according to personal finances
Serious injuries over time or not	-Injury over time	-No serious injury over time
Concerns about the future and the need for education	-Expectations for the future -Strong desire to study -Difficult to combine study and football	-Have completed the education -Live as full-time professional
Trust from the coach and playing time	-Lack of trust from the coach and playing time -Unhappy with the coach	-Lots of trust from the coach and playing time -Feeling of mastery -Joy of playing and developing oneself
Prioritizing football, or not	-Other interests than football -Other priorities	-Few other interests than football -Football as a priority

## Results and Discussion

The analysis revealed six main factors, as presented in Table 1, influencing whether players end or continue with elite football. These will be presented in more detail and further discussed.

### Extent of Internal and External Motivation

The analysis shows that motivation is a factor mentioned by all informants, in respect of both career ending and career continuing. Lack of motivation for using time, energy and money on football clearly influences the decision to end the football career. Similar findings are presented in the study by Brandt-Hansen et al. (2014), where lack of motivation was one of the three most important reasons for drop out from the elite level among Danish women players. Whether this concerns internal or external motivation is difficult to ascertain from the interview data, but it seems reasonable to suppose that both play a part in both career ending and career continuance.

The analysis showed little evidence of internal motivational factors among the elite players who ended their football career at a young age. A majority of the elite players described the motivation to play football diminishing throughout their career as they experienced life as an elite player becoming heavier and tougher, and they felt more and more that it came at the cost of other things. In the quote below, Andrea describes lack of internal motivation as a factor that seems to set her apart from those who still play elite league football:

*[…] football is, really, not my whole life, even though I love it and like to be part of a team, I have so many other things that make me happy as well. […] So, I think that, in a way, I've not completely had that inner drive, and that has made me not want to commit 110%. I've gone – like - to see friends and gone to a party because that's how I am. I've gone on a cabin-trip rather than stay at home and get myself ready for a game in a fortnight's time*.

Concurring with Andrea's view, Berit also describes the large part that lack of internal motivation played in her decision to end her football career. She felt that motivation for other things was greater than motivation for football. To the question about why she, Berit, had chosen to end her career, she answered:

*It was the training really that did it. And having to keep free those regular times outside of working and studying. So, it was really freedom that I wanted to feel, I think – to be able to decide for myself what to do with my evenings and not have to say no to so much*.

In contrast to these quotes, elite footballers who continue to play displayed a high level of internal motivation through their eagerness to play, as Frida says:

*I actually decided when I was about 12 that I would play for the national team and set myself a goal then to do so. I've always had that eagerness to be able to play football and somehow make that happen*.

In addition, elite players described the inherent joy of playing the game itself and the training, as Gina thought, when she described what the main reason is she still plays elite football:

*If I will say one thing … […] Then joy. I like it so much. I'm a person who plays football and trains and finds joy in it*.

This finding is supported by the study of Brandt-Hansen et al. (2014) where joy in the game, the joy of performing for yourself and the possibility of improving oneself, appeared to be the most important motivational factors for female Danish footballers at elite level.

### Financial Circumstances and Total Load Level

The analysis showed that financial circumstances were a factor of significant importance, for both career ending and career continuing elite players. Danielle makes clear the issue of lack of wages:

*You can't really go through the whole of your 20s and start of your 30s with what is far below the minimum wage in Norway. It demands a lot of motivation and hard work if you are to carry on without a salary you can live on, and at the same time retain the possibility to doing well in studies, and in football as well*.

Of the players who had ended their elite football career, most had combined football with work when they were active. In addition, three of the five former elite players had studied whilst they played football. Gjerset et al. (2015) defines total load as the sum of the load from trainings and competitions, as well as other mental and physical strains the athlete is disposed of in an everyday life. In this study, the informants focus on work and studies as other physical strains that have an impact on the total load. Former player Cecilia describes the stress of devoting so much time and energy to work and earning a living.

*The help that you might have received that would have made it possible to carry on a bit longer would of course, playing at the top level, have been some kind of financial compensation. Had you been able to cut one thing, that you had to work part time beside playing football and full-time study. […] Compensation has mostly to do with that, I think that some financial support could have helped many of us. I know that with the friends I have who earn a little bit, that they then can at least earn a little so they can have a somewhat smaller job outside football and just that helps. That they can work 80% instead of 100%. I think that would have helped*.

Unlike the elite players who have ended their football career, the players who continued their career have been offered better pay and conditions from the club, as Gina explains:

*I understand why so many give up. When I look back, if I had gone up in pay to the level I have now, I would have had to have thought about it too. Because for a long time it didn't really work*.

Of the nine elite players who took part in the study, only two lived as full-time professionals. Four received, or had received, a wage from their club but this was not enough to live on without a second job or a study loan from the state. There is much that suggests that our informants were representative of women footballers in Norway. Several, however, made the point that considerable change has occurred during their career. They describe financial developments in recent years associated with the overall development of women's football in Norway. This is supported by Balsvik (2019), pointing to a process of positive change in Norwegian women's football during these years, not least increased sponsorship after 2017. Balsvik describes the positive effect of the commercialization of, and investment in, women's football in Norway during the past few years. On these grounds, there is reason to suggest that those elite players who had ended their football careers in 2016 or earlier, probably did not have the same financial starting point as the still active elite players:

*[..] if there could have been just a bit more money or flexibility, I think it would have made things a bit easier, and easier to carry on. I felt very strongly that I simply must work to earn some money because you just didn't earn enough from football. […] Of course, you had bills needing to be paid, I was terribly aware of that (Jessy)*.

The analysis suggests that these financial concerns and expectations were not an issue earlier in the player's career but grew with time. When asked what impact finances had had on her football career, Jessy answered:

*Not much, but mostly because I gave up when I was 21. So, it didn't have the greatest effect on me. When you're still at school, living at home, get the cost of your kit covered and have four or five thousand a month, that's completely OK. In the USA as well pretty much everything was covered, but when you're studying or doing other things, four or five thousand is nothing. So, it would have affected me had I carried on. But no, when you're 17 or 18 and living at home it doesn't matter much*.

Jessy's view was supported in the interviews with the players who were continuing their career. These players currently have an average age of 27.8 and confirmed that with increased age come financial anxieties. This may, therefore, indicate that the need for money, and worries related to finances, often come at an age where you become more independent of your parents and in connection with moving away from home and establishing yourself. This was also the understanding of Brandt-Hansen and Ottesen (2017). Of the 102 elite footballers taking part in their study, the majority were in the age range 26–32 and had a full professional contract. This shows a clear tendency for both the possibility of and need for a full-time contract to increase with age. That 41% of these elite players still lived at home with their parents shows that their need for financial support was not as great as that of elite players living on their own and having greater financial outlay (Brandt-Hansen and Ottesen, 2017). In her answer, Gina confirms that the need for better financial circumstances increases with age.

*It works at a certain age, and it works for a time. As I say, when you're 19,20,21,22, then you can be grateful for the little you have. But then comes a time when you just need more. And if you don't get it, then all that you have got from football that has counted until then, doesn't add up anymore. Other things have their own weight. You want to manage on your own, get yourself a job and cope with daily life without being worried. Because those worries cost a lot of energy. So, I think many have experienced that*.

Even though all the elite footballers still playing received either full or partial support from their club, there were several who nonetheless encountered financial difficulties at certain points in their career. Gina expressed her thoughts about, and experience of, these tough times in the following way:

*It's a question of whether it works at all. Having to pay for food, petrol, bills without being anxious. Because there were quite a lot of years when I was anxious. There was one time when I just went to pieces, because it was like, “I don't have the means, I don't have any money.” I don't like it being like that, it's awful*.

Kaelberer (2019) concludes that the interaction of sports associations and clubs, business sponsors and the media, prevents women's football from effectively challenging hegemonic masculinity in German football. This partly because <10% of newspaper coverage or television broadcasting is devoted to women's sport, he argue, and states that to work against hegemonic masculinity requires greater societal and popular support.

However, our findings also show that the interests in female elite football increases, and the financial circumstances among female elite players are getting better. This is in accordance with Kaelberer (2019), who claims that female football players have made some forays into a previously exclusively masculine preserve. He highlight that more people pay attention to female football, and that the earnings for female football players have increased. Furthermore, female football players have started exploring new forms of femininity that include masculine traits such as self-confidence, athleticism and muscularity.

Despite many female elite players in Norway having similar experiences and maybe needing a job or qualification alongside football, it is reasonable to believe that financial developments within women's football in Norway in recent years have affected female elite players' financial circumstances. This development is confirmed by those players who have continued their career when they talk about the comparison between today and earlier, describing, now, a feeling of financial security, either as fully-fledged elite players or with an optimal combination of salary from club and job or study. They point to the introduction of “professional days” as representing a significant improvement. This is an arrangement in which the clubs receive support from *Toppfotball kvinner* (the League Association for Women's Football) to cover the wages of players having days off work to be able to train earlier in the day, and train more during the day. Frida describes this below:

*No, it was when they began to introduce professional days amongst other things. It meant that we could begin to train earlier. And, of course, that we have the stadium, our own pitch, own changing room, somewhere to eat, could wash clothes. Little things like that. If it had been men's football, it would have meant little to them, but it makes such a difference for us. Just being able to wash clothes and get an evening meal once a week. Little things that left us pleased*.

Our informants' descriptions of changes in the economic conditions within women's football are in keeping with the findings of the study by Wang (2019). Our findings, however, contrast with those of Brandt-Hansen et al. (2014), who found that neither money or other material benefits had particular significance for the motivation and ambition of elite female Danish footballers. One reason why finances are proving to be more important for the motivation of the elite Norwegian female players in our study may be the increasing prominence of women's football in recent years. It is natural to suppose that the expectations of these players would rise in line with this development, and the possibility of earning money is significantly greater now than when Brandt-Hansen et al. (2014) undertook their data collection. On the other hand, the opportunity for elite female players to earn more money can lead to more expectations and commitment to football. A consequence of this can be less time to study, having an outside employment, or doing other things beside playing elite football. It could therefore become more difficult to get time to take an education and having this as a security after ending the football carrier. For two of the elite players who ended their football careers at a young age, they had other interests and other priorities alongside football. Expectations to prioritize football will naturally be bigger when the players are full-time professionals, than when they are semi-professionals. However, it is not given that all women football players want to be a full-time professional.

According to this finding, we will argue that this development is actually challenging the hegemonic nature of football, which is important according to Kaelberer (2019), and making football an arena for more than the performance of hegemonic masculinity. Although, for many elite women footballers, a job or training in tandem with football will be necessary for them to survive financially. An understanding of female elite players' total burden can be closely linked to the term “dual careers” used to illustrate the demands placed on elite British female players. Like the British players in the study by McGreary et al. (2021), our analysis shows that the Norwegian elite players in this study experienced similar challenges in having dual careers. For those players who had ended their career, financial worries and hence a high level of stress had had an indirect effect on them quitting their football careers. This finding is consistent with Grygorowicz et al. (2019), who found that too little time for training due to the demands of work and study was the commonest reason for the Polish players who participated in the survey giving up their football careers.

Through the analysis, it emerged that the lack of pay in women's elite football created a challenging situation, entailing a lot of work and/or study, and thereby a high total load, including for the elite players who still played at top level. This also emerges from the interview with June, who now gets her income through a professional contract - something she did not have before:

*If I compare it. It's only now that I realize how difficult it was. I went to college. Cycled there and was there until three at least and then cycled straight to training with team meetings and a quick hello. So, I got home, ate dinner and went to bed. So, it was very hectic and a high total load. I noticed that I didn't get either enough food or drink and very little rest and recuperation. While now it's almost like I don't quite know what to do with myself during the day, but I at least get time to recover and get enough sleep and feel that my body is more in balance as a result. That's really how it should have been for many more people*.

In summary, our findings indicate that a challenging financial situation and high levels of stress factors contributing to the elite players ending their football career. At the same time, these were also factors mentioned by the elite players who still play football when asked about possible reasons why they have considered ending their football career. In a study, Lie (2017) show that men on the national football team earns an average of 640,000 Euro more a year than a woman on the national football team. These differences are also clearly shown in our study, as the majority of the informants lived as semi-professional players and gave the impression that they are not or have not been well paid from the football club they played for.

### Serious Injuries Over Time or Not

The analysis showed that a long injury layoff was a factor mentioned in relation to several players who had dropped out. Cecilia, Danielle and Jessy, who had all ended their football career, had suffered severe injuries which would have required long rehabilitation in order to return to full fitness. Each had suffered a different injury, but the common denominator was an uncertainty related to the duration of the injury, whether they would be completely fit afterwards, and the fear that they could injure themselves again. All three had had future plans for their football careers, with Jessy still possibly a member of the national team and Danielle and Cecilia having just changed clubs. Asked whether they would have continued their career had they not been injured, all three answered that they would most likely have played on for some years. Despite all three describing several factors as contributing to their retiring, the injury seemed to be the triggering factor. Jessy experienced that her cruciate ligament snapped for the second time during her football career:

*[…] ..and, after that, I trained hard and was ready to play again, but I knew that my motivation had gone down with the second knee injury, and I had no wish to see it happen again. And there was something to do with finances on top of all that as well. As a woman footballer in Norway, it hasn't, in a way, been profitable. And although I'm not alone in that, I've had to work alongside the football the whole time*.

This agrees with earlier research into cruciate ligament injuries in footballers. According to Sandon et al. (2015), there is a greater risk of cruciate ligament injury for women than for men. There are also fewer women than men who have returned to football after such an injury. One reason for this may be that male players have a job offering good financial provision, while the majority of female players are semi-professionals without such good conditions (Gammelsæter and Solenes, 2013). This understanding is emphasized in the interviews with Jessy and Cecilia, who say that the injury, taken together with the financial strain, was the reason they chose to end their football career. The financial strain was not an issue for the third player who chose to end her career, as she was living at home at the time.

All the players still active in elite football said that they have suffered minor injuries during their career but described themselves as fortunate not to have suffered severe injury leading to a long layoff. Of the four still playing football, only Gina said that injury had affected her career. She has struggled with stress injuries and played many games while in a lot of pain. However, she has felt that the team depends on her, and has therefore been favored by the coach despite her injuries. Based on this, it can be argued that none of the elite players who still play football have experience of “time-loss injuries”, as they have not been away from training and matches for long periods due to injury (Fuller et al., 2006).

### Concern About the Future and Need for Education

The results show a contrast between players who have ended their career and those who have continued theirs, in terms of anxiety about the future. On the one side, were three out of five elite players who described worries about the future as being a factor contributing to them ending their football career. On the other hand, none of the elite players who have continued their football career conveyed any sense that they thought of concern about the future as a factor that might make them give up.

A summatory finding of the analysis is that all five elite players ended their career whilst engaged in gaining qualifications. They all said that the combination of playing and studying was demanding, and that it influenced their decision to end their career. Several made the point that the motivation to study was greater than that to continue playing football, as Cecilia says:

*[…] In my case, I knew that studying was more important than the football, which meant that for me it was not enough to play football and try to rely on that, and work part time somewhere just to get an income. I had always known that I would study and knew what I would do, and that it was more important to me, plain and simple*.

What Cecilia said agrees with what players have said in several other studies. McCormack (2011), Gammelsæter and Solenes (2013), and Brandt-Hansen and Ottesen (2017) all emphasize, in their studies, the importance education holds for elite female players. The reason for this is often held to be that the future prospects of female elite players, compared to male elite players, more often involve less accumulated funds and thus a more uncertain financial future. For two of the former elite players in this study, Berit and Danielle, it was precisely expectations of the future that led them to enter into a course of study. After she found it impossible to combine her studies with football—which was a contributory factor in her decision to end her elite playing career—Danielle explained as follows:

*As a female footballer, you live in the knowledge that you are engaged in something that, for everyone, or nearly everyone, will not be possible to live on. This was, in a way, the background to my beginning to study. And being a student was a lifestyle not really compatible with being a top sportswoman. If the conditions for being a footballer had been better, then perhaps I would have carried on*.

Of the elite players who were still active, three out of four had completed higher education. They had, therefore, succeeded in combining their football career with acquiring qualifications. Presently, only one of them uses her qualification and combines having a job with football. The others live either completely from football or receive an income from a scholarship or loan in connection with their studies.

*I don't earn much from football, and I can't really just play football. There are lots who give up for that reason, so therefore I've taken a qualification as well and I'm really happy to have that. […] So, I think there's loads that needs to happen. It has to be made to work so that income stops being a factor. Now, I'm not scared for the future, in a way, because I have my qualification. I think there are lots who stop playing because it's difficult to study whilst you are playing football, or because they can't manage to combine football with a job (Heidi)*.

Like Heidi, June and Gina managed to combine football and studying. The grant that came with the course was for her a solution making it possible for her to continue playing football.

Of the four elite players still active, three said, however, that they had considered stopping on one or more occasions. On this topic, they spoke of anxiety about the future, particularly in relation to having sufficient funds to provide for themselves. Frida was the only one who neither had a qualification outside football nor was working toward one. For her, qualifications were not something she thought of as being necessary given the life she was living. One reason for that may be that she had a full professional contract and was therefore not dependent on a grant or loan as Gina was. This chimes with Gammelsæter and Solenes' (2013) argument that fully professional players see themselves more as being employed and are therefore more willing to delay qualification.

### Trust From the Coach and Playing Time

Analysis of the interview material indicates that trust from the coach and playing time are related to the motivation to play elite level football. Among the elite players who have ended their career, little playing time had had a negative effect on their motivation, whilst playing time and the trust form the coach that came with it had a positive effect on those players who felt that they were being relied on.

Andrea and Berit described playing time and the coach's trust in them as being central factors in their having lost the motivation to play, which in turn led to them giving up. Both of them were regularly in the squad for both home and away games but started the majority of games on the bench and generally got little playing time. For them to be match fit, they were therefore required to play in second team games, often the day after the first team game. They describe, therefore, a very high level of pressure involving a lot of traveling, which again affected their motivation. Andrea describes the feeling she had when she had little playing time and little belief in her from her coach in the following way:

*[…] So, I go there and bash myself on the head on Monday, Tuesday, Wednesday, Thursday and Friday to be good in training. And then you go there on Saturday and Sunday and think: “OK, now I'll be good when I get on the park.” And then you don't get to play, and you become really disappointed and angry, because you have to start all over again the next week. So, you never get that affirmation. When you don't get it in training either, it doesn't help*.

In a study by Johansen (2016), female players stressed the need for the coach to see them, and for them to get the recognition they needed to perform in training and during the match. There was also shown to be a connection between the amount of playing time they got and the trust showed by the coach, and their attitude to the coach. These are signs of this in our study as well. The players who had ended their career because of little playing time expressed displeasure with their coach and had clear opinions about how things should have been done differently. Brandt-Hansen et al. (2014) found, in their study, that the factors: “football is fun”, “the joy of performing” and “always possible to improve” were the most important motivational factors in Danish footballers playing elite level football. Loss of confidence as a result of little playing time leads to there being little pleasure, progress or performance for players, the result can be that they drop out. Andrea, a former player, elaborates on this:

*How much you get to play depends on the coach's belief in you. If the coach trusts you and you do a good enough job, so you get to play. And if you are playing, then you most likely have a lot more fun. You don't become a footballer to sit on the bench. Everyone knows that. That's no fun at all, it's the match that's fun*.

What characterizes the elite players who have continued their football career beyond the average age, is that when they were first included in the first team at a Norwegian top league club, they gained the trust of the coach which in turn resulted in a lot of playing time. None of them had had to spend much time since then on the bench for their team, and all have been involved in age-specific national teams or the national first team throughout their footballing career. Based on this group of elite players still playing football having had so much playing time, the trust of their coach and, additionally, a background in national teams, there are many indications that these players have maintained a high elite level and been among the very best. Even though women's football has come a long way in recent years, women footballers are still, by Gammelsæter and Solenes' (2013) definition, categorized as semi-professionals. Only a minority, therefore, live as full-time professionals and, naturally, it is elite players who are seen as being the best. That is, the eye of the needle is narrow and the probability of being among the best small, and thus the uncertainty regarding the future as a female elite player is great. The connection between a national team background and perceived trust and support from the coach is emphasized in the study by Brandt-Hansen et al. (2014). According to their survey, there was a significant difference in perceived support and trust among those who had a national team background, compared with those who had never been in the national team. In the present study, former elite player Andrea expressed a similar view, and implied, indirectly, that coaches often treated those in the national team and those who were not differently. When asked about what difference it would have made to her career had she been in the national team, she answered:

*Well then would get the affirmation you need. And I think that all the trainers I've had – a strong word this – adore all those who've been in the national team. As if playing for your country differentiates those who are good from those who aren't*.

### To Prioritize Football or Not

The analysis shows that what characterized the elite players who had had little playing time in their football career, was that they had more interests which they prioritized over football, in comparison to the active elite players. There are some indications that Danielle and Andrea, who chose to end their elite football career, had interests outside of football that may have made it easier for them to choose to leave elite football. This becomes clear in the interview with Danielle when she was asked what factors influenced her to end her football career.

*If I compare myself to others, so I have more outside interests. They don't have them […] I think that's because I have something to turn to after football, and it's been fine without football. I don't think it would have gone as well if I hadn't had it. I think I'd have struggled more*.

Andrea's answer to the same question was almost identical to Danielle's. In contrast to the elite players who had ended their football career, all the players who continued their football career said that football was their first priority, even at the expense of time with friends, parties, social events and other interests. This was clearly expressed by Frida:

*Football has always been, has come first. It takes priority. It's clear that sometimes, really, I don't feel it so often, that I should have been at this party or […] It's clear that I don't get to see my best friend so very often, but no, it's not been that hard. Sometimes it's difficult living away from family and so on, but it's always been fine. It's been easy to prioritise*.

The above finding is in agreement with the results of Brandt-Hansen et al. (2014).

### Strengths and Weaknesses of the Study

The results are derived from in-depth interviews with five elite players who have ended their football career at a young age, as well as four elite players who have continued their career beyond average age. Taken together, the informants represented a wide range of elite players who have played and are playing in the Norwegian top league, and who have backgrounds and experience from various clubs around Norway. The findings are substantiated to a large extent by previous studies, but also add new knowledge. To be a qualitative study using in-depth interviews, the study includes an acceptable number of participants. Furthermore, that most participants seem to confirm the same main findings, strengthens the credibility and reliability of the study. However, due to the small number of respondents, it is necessary to be cautious about the generalizability of our conclusions. This might require quantitative studies, including statistical analyses. Nonetheless, the findings have a credible general validity on the basis that the participants seem to highlight the same factors as important according to drop out, or continuation, with elite football, and that all people involved in women's football have something to learn from these female players' experiences.

## Conclusions

The results of this study indicate that factors leading to elite players ending their football career at a young age are; low level of internal and external motivation, poor financial circumstances leading to high levels of stress, long absence through injury, concerns related to education, lack of playing time, and prioritizing other activities than football. Still active elite players reported higher level of internal and external motivation, better financial circumstances in football—and thereby lower levels of stress, no serious injuries and fewer concerns related to education, lots of trust from the coach and playing time, and also prioritizing football over other activities.

On the basis of the analysis, there emerged a clear distinction between the level of internal and external motivation between the elite players who have ended their football career and those who have continued theirs. External motivation in the form of benefits such as wages and recognition were absent from the career of those elite players who had stopped playing. For the elite players who were still active, expectations of better salary and perceived recognition were motivations to continue their football career. Low internal motivation to return from injury, or to play their way back into the team, were indirect reasons for elite players ending their football career. For the elite players who chose to continue their career, on the other hand, inner motivation in the form of the joy of playing football was emphasized as being absolutely crucial for them in continuing their football career. Another main finding was that long-term injuries and lack of playing time were underlying reasons for elite players ending their career at a young age, and that long injury breaks and lack of playing time seem to lead to the reduced priority of football. On the other hand, freedom from injury and ample playing time appear to give football first priority, and thereby lead to a longer-lasting football career. A key finding in the study seems to be that female elite players today feel that their financial circumstances are improved, in comparison to a few years ago. For those who ended their career, the high level of total load appears to have played a decisive role in their decision. The elite players who were still active, earned better through football, had had fewer injuries, experienced being relied on in terms of playing time, gave football high priority and had a greater degree of internal and external motivation. In sum this constituted a lower total load enabling a greater investment in football, which in turn prolonged their career.

This study is giving new insights in women's football in Norway, that is a minor studied field in both Norway and internationally. Because of few researches on women and football, this study will be important to put the focus on women football and hopefully lead to more studies in this field of research. The study reproduces findings in other studies according to early drop out. New insight in this study shows is that establishing a family and having children not is necessary a reason for early dropout from elite football. None of my informants mentioned this as a factor that influenced the choice of dropping out of elite football at a young age. This study also shows the importance of getting enough playing time, even if it's because of a serious injuries or lack of trust from the coach. All five informants who left elite football at a young age emphasized that it was because of lack of playing time. This article indicates that the framework conditions for female elite players are about to change in step with the development of women's football both in Norway and internationally. Both better finances and less total load are described as better today than it was when the informants started their football careers. They describe an everyday life that is gradually becoming more and more professional and makes it easier to invest in a football career. This qualitative study contributes to a deeper understanding of factors that influence female elite players' football careers. Based on the fact that the study has few informants, the results as previously mentioned cannot be generalized to larger groups. Further research should therefore focus on a quantitative approach that will include a larger sample. In summary, I would argue that future research on women's football is necessary for girls and women to have access to relevant findings that can help improve their conditions in the future and hopefully help their football careers last longer. According to Barreira (2016) the age of the elite players was related to their performance. Getting female elite players to extend their football careers can therefore help to develop even better female football players in the future.

## Data Availability Statement

The raw data supporting the conclusions of this article will be made available by the authors, without undue reservation.

## Ethics Statement

The studies involving human participants were reviewed and approved by The Norwegian Centre for Research Data (NSD). The patients/participants provided their written informed consent to participate in this study.

## Author Contributions

All authors listed have made a substantial, direct, and intellectual contribution to the work and approved it for publication.

## Conflict of Interest

The authors declare that the research was conducted in the absence of any commercial or financial relationships that could be construed as a potential conflict of interest.

## Publisher's Note

All claims expressed in this article are solely those of the authors and do not necessarily represent those of their affiliated organizations, or those of the publisher, the editors and the reviewers. Any product that may be evaluated in this article, or claim that may be made by its manufacturer, is not guaranteed or endorsed by the publisher.
